# Folic acid use in pregnant patients presenting to the emergency department

**DOI:** 10.1186/1865-1380-4-38

**Published:** 2011-06-24

**Authors:** Jacob Steenblik, Erika Schroeder, Burke Hatch, Steven Groke, Camille Broadwater-Hollifield, Michael Mallin, Matthew Ahern, Troy Madsen

**Affiliations:** 1 University of Utah, Salt Lake City, UT, USA; 2 Division of Emergency Medicine, University of Utah, 30 N. 1900 E. 1C26, Salt Lake City, UT 84098, USA

## Abstract

**Background:**

The US Preventive Services Task Force has recommended daily folic acid supplementation for women planning on becoming pregnant in an effort to prevent fetal neural tube defects. We evaluated pregnant patients presenting to the emergency department to determine rates of folic acid supplementation.

**Methods:**

We surveyed a convenience sample of pregnant patients who presented to the University of Utah Emergency Department (ED) between 1 January 2008, and 30 April 2009, regarding pregnancy history and prior medical care.

**Results:**

One hundred thirty-five patients participated in the study. Eighty-four patients (62.2%) reported current folic acid supplementation. Sixty-six patients identified themselves as Caucasian and 69 as non-Caucasian race. There was a significant difference in folic acid use between Caucasian and non-Caucasian women (*p *= 0.035). The majority of Caucasian women (71.2%) reported daily folic acid use versus approximately one-half of non-Caucasian women (53.6%). Both groups were similar in accessing a primary care provider (PCP) for pregnancy care prior to the ED visit (53% vs. 49.3%, *p *= 0.663), and rates of folic acid use were similar in those who had seen a PCP (85.7% vs. 76.5%, *p *= 0.326). Language did not have a significant association with folic acid use.

**Conclusion:**

A large percentage of pregnant ED patients did not report current folic use, and there was a significant difference between Caucasian and non-Caucasian women in rates of folic acid supplementation. This study highlights the potential role of the ED in screening patients for folic acid supplementation.

## Introduction

In the United States, approximately one in every 1,000 pregnancies is affected by a neural tube defect (NTD) [1]. Among the most common types of NTDs, spina bifida and anencephaly are estimated to affect approximately 3,000 pregnancies each year in the US [2-7]. In an effort to curtail these preventable birth defects, the Food and Drug Administration (FDA) has suggested that women of childbearing age consume a minimum of 400 μg (0.4 mg) of folic acid daily [1,2,8-10]. Despite these recommendations, total folate consumption remains well below the recommended levels in Hispanic communities when compared to non-Hispanic communities [1-5,8,10]. Additional research has suggested that non-Caucasian females of child-bearing age are significantly less likely to take a prenatal vitamin [10].

Pregnant patients presenting to the emergency department (ED) may represent a higher risk group that is less likely to have received prenatal care or appropriate education regarding folic acid supplementation [11]. Emergency departments have been successful in performing nursing screening and intervention for domestic violence prevention and alcohol abuse, and may represent an appropriate setting for intervention regarding folic acid supplementation in pregnancy [12,13].

We sought to evaluate the rates of folic acid use among pregnant patients presenting to the ED. Furthermore, we intended to identify patient characteristics related to folic acid use in an attempt to potentially define the role of the ED in screening for folic acid use and aiding in the prevention of neural tube defects.

## Methods

We conducted a survey study of a convenience sample of pregnant ED patients over the 16-month period from 1 January 2008, through 30 April 2009 at the University of Utah Medical Center ED in Salt Lake City, Utah. The University of Utah ED is an urban, academic ED that treats approximately 39,000 patients per year.

All pregnant patients in the ED were eligible to participate. Patients were approached by trained research associates and asked to complete a survey regarding their pregnancy history and current medications. All pregnant patients who participated in the study had presented with pregnancy-related complaints of abdominal pain and/or vaginal bleeding. Research associates were present in the ED 7 days per week from 8 a.m. until midnight.

The survey consisted of questions regarding pregnancy history, current medications, and prenatal care. Patients were specifically asked the question, "Are you taking prenatal vitamins?" We utilized this question as we felt the term "prenatal vitamins" would be the term most familiar to patients to describe a folic acid-containing supplement.

Patients were asked to self-identify their race and primary language with free-text spaces in which the patient recorded this information. Utilizing self-reported race, patients were grouped according to reported race using United State Census classifications [14]. In evaluating rates of folic acid use, comparisons were made between patients reporting Caucasian race and those reporting a race that was not Caucasian (non-Caucasian). In two cases, patients reported "multi-racial" as their self-reported race. For statistical analysis, these patients were classified as non-Caucasian.

Patients were asked about their medical care during the pregnancy with the following question: "Have you been to a health care professional (OB, family practitioner, midwife) for care during this pregnancy?" Patients were asked to report the date of the first day of their last menstrual period, and gestational age was calculated based on this. The survey was one that we developed and that underwent internal revision and validation without an external validation processes (see Appendix).

Chi-square testing and Fisher's exact test were used for categorical variables (SPSS v. 16.0). For associations, we report *p*-value, odds ratios, and 95% confidence intervals (CI). This study received approval from the University of Utah Institutional Review Board (IRB).

## Results

One hundred thirty-five pregnant women participated in the study during the 16-month period. Of these, 23 patients (17%) presented with vaginal bleeding, 44 patients (32.6%) presented with abdominal pain, and 68 patients (50.1%) presented with both vaginal bleeding and abdominal pain. The average age of the patients was 25.1 years (range 16-42 years). The average number of pregnancies per patient was 2.8 (range 1-9) with an average of 1.1 previous live births per patient (range 0-6). The average estimated gestational age was 73.2 days (range 13-147 days) (see Table 1).

**Table 1:** Patient Characteristics

Characteristic	Number/Percentage (Range)
Total Patients	135

Average Age	25.1 years (16-42)

Number of Pregnancies	2.8 (1-9)

Previous Live Births	1.1 (0-6)

Average Gestational Age	73.2 days (13-147)

Non-Caucasian Race	51.10%

Current Prenatal Vitamin Use	62%

**Figure 1:**
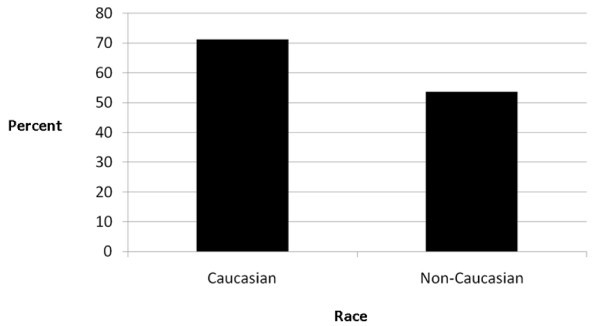
**Prenatal vitamin use by self-identified race**.

Of the women, 62.2% reported current use of a prenatal vitamin at the time of the ED visit. We noted a significant difference in prenatal vitamin use between patients who identified themselves as Caucasian and those who self-identified as a non-Caucasian race. Sixty-six patients (48.9%) identified themselves as Caucasian, while 69 patients (51.1%) identified themselves as of non-Caucasian race. Patients who self-reported a non-Caucasian race identified themselves as follows: Hispanic (68.1%), African American (11.6%), Native American (7.2%), Pacific Islander (5.8%), Asian (4.3%), and multi-racial (2.9%); 71.2% of Caucasian women reported prenatal vitamin use compared to 53.6% of non-Caucasian women (*p *= 0.035, OR = 2.14, 1.05-4.36) (see Figure 1)

Caucasian and non-Caucasian patients were similar in reporting having seeing a primary care provider for prenatal care prior to the ED visit (53% vs. 49.3%, *p *= 0.663, OR = 1.16, 0.59-2.28). Among those who were seen by a primary care provider (*n *= 69), rates of prenatal vitamin use were similar (Caucasians: 85.7% vs. non-Caucasians: 76.5%, *p *= 0.326, OR = 1.85, 0.54-6.35). Language did not have a significant association with parental vitamin use. Of the patients, 25.9% identified their primary language as a language other than English. Sixty-six percent of patients who stated their primary language was English reported prenatal vitamin use vs. 51.4% who identified their primary language as a language other than English (*p *= 0.126, OR = 1.83, 0.84-4.01).

## Discussion

Approximately 70% of neural tube defects could be prevented with the consumption of folic acid before conception and in the early stages of pregnancy [5,8-10]. As we have reported in our study, a significant percentage of pregnant patients presenting to an emergency department did not report current folic acid supplementation. Perhaps even more notable, this difference was particularly pronounced when comparing patients who self-identified as a non-Caucasian race. Previous studies have noted a more significant dietary deficiency of folic acid in non-Caucasian patients [1,5,8,10].

The reason for lower rates of folic acid supplementation in non-Caucasian patients in our study does not seem to be related to language barriers leading to misunderstanding or failure to receive appropriate guidelines for folic acid supplementation. We found that those who identified a primary language other than English were not significantly less likely to reports folic acid supplementation. This difference also did not seem to be related to discrepancies in the prenatal care provided between Caucasian and non-Caucasian patients who saw a physician prior to their ED visit. Of those who had reported visiting a health care provider prior to their ED visit, folic acid supplementation was similar between these two groups.

Given these findings, this study suggests a potential role for the ED in the prevention of NTDs. As this study demonstrates, a high percentage of pregnant patients are not currently using folic acid supplementation. Folic acid supplementation education delivered in the ED may be a viable public health initiative. This intervention could be as simple as screening childbearing-age women for folic acid use and providing an information sheet on the benefits of folic acid supplementation, as well as a list of community resources available to these patients.

While our study suggests the potential for a folic acid intervention program, it does not suggest that such an intervention would be effective in improving the rates of folic acid supplementation among pregnant women. Other ED-based intervention programs, however, have previously proven effective. Examples of these include screening for domestic violence and alcohol abuse [12,13]. Further research would seem indicated to determine the efficacy of an ED-based folic acid supplementation intervention.

## Limitations

This study did not evaluate the outcomes of the study participants to determine pregnancy complications for those who did not report folic acid use. For many of the patients we evaluated, it is likely that initiation of folic acid at the time of their visit may have been too late to prevent neural tube defects, given that the range of reported gestational age extended to 147 days.

As a convenience sample of patients, this does not represent all pregnant ED patients during the study period, and thus may not adequately represent folic acid use in this population. The convenience sample is subject to bias based on both the availability of research associates as well as patient willingness to participate in the survey. Thus, this may not adequately represent folic acid use among the full spectrum of pregnant ED patients.

Similarly, our study focused only on those patients who presented to the ED with pregnancy-related symptoms. Ideally, folic acid supplementation would be initiated by all women of childbearing age. Although we presume that the rate of folic acid use in this larger population would be even less than in the pregnant patients we studied, we are unable to draw conclusions related to this population from the information we have gathered.

It is unclear whether low rates of folic acid supplementation may have been unique to our catchment area, or whether these lower rates, particularly among non-Caucasian patients, may have been due to characteristics of the primary care network or health care initiatives in our region. Similarly, while we did not find that language was significantly associated with differences in folic acid supplementation, language barriers or cultural differences between patient and physician may affect the efficacy of physician counseling and recommendations. We have not specifically evaluated the multiple questions related to the provider-patient dynamic and how this may have affected the results that we noted in our study.

## Conclusion

Neural tube defects may be prevented through folic acid supplementation in early pregnancy. Our study demonstrated both a low rate of folic acid supplementation among pregnant ED patients and a significantly lower rate of folic acid use among non-Caucasian patients. This study suggests the potential need for an ED-based educational intervention program as a means to improve folic acid supplementation in pregnancy.

## Competing interests

The authors declare that they have no competing interests.

## Authors' contributions

TM, ES, BH designed the study. TM, JS, ES, BH, MM, CBH, and MA contributed to the data analysis and review. TM, JS, ES, BH, MM, CBH, and MA provided significant contribution in the writing and revision of the manuscript.

## Appendix

### Emergency Department Pregnancy Study Questionnaire

Please tell us about yourself:

Name:

Telephone number:

E-mail address or alternative phone number:

Primary language:

Age:

Race:

Please tell us about your previous pregnancy history:

How many times have you been pregnant?

How many times have you given birth?

How many times have you had live births?

How many spontaneous failed pregnancies or miscarriages have you experienced?

How many induced abortions have you experienced?

How many Cesarean deliveries have you experienced?

Have you ever been hospitalized for treatment of pelvic inflammatory disease? Yes No

Have you ever been treated as an outpatient (out of the hospital) for a pelvic infection from chlamydia or gonorrhea? Yes No

Have you ever used an intrauterine device (IUD) for birth control? Yes No

Have you ever had pelvic surgery (not including cesarean section)? Yes No

Please tell us about the symptoms you have experienced recently:

What was the date of your last menstrual period?

Have you had an ultrasound to evaluate your pregnancy prior to this visit? Yes No

If so, what is your estimated delivery date based on this ultrasound?

Have you been to a health care professional (OB, family practitioner, midwife) for care during this pregnancy? Yes No

Are you taking prenatal vitamins? Yes No

Are you taking aspirin? Yes No

Do you smoke? Yes No

Please list any other medications you are currently taking:

How much pain are you experiencing? (please circle one of the choices below)

None (no pain)

Mild (less than your menstrual period)

Moderate (equal to your menstrual period)

Severe (more than your menstrual period)

How much vaginal bleeding are you experiencing? (please circle one of the choices below)

None (no bleeding)

Mild (less than your menstrual period)

Moderate (equal to your menstrual period)

**Thank you for your participation in this study**.
